# PI4KIIα regulates insulin secretion and glucose homeostasis via a PKD-dependent pathway

**DOI:** 10.1007/s41048-018-0049-z

**Published:** 2018-03-07

**Authors:** Lunfeng Zhang, Jiangmei Li, Panpan Zhang, Zhen Gao, Yingying Zhao, Xinhua Qiao, Chang Chen

**Affiliations:** 1 National Laboratory of Biomacromolecules, CAS Center for Excellence in Biomacromolecules, Institute of Biophysics, Chinese Academy of Sciences, Beijing, 100101 China; 2 University of Chinese Academy of Sciences, Beijing, 100049 China; 3 Shanghai Institute for Advanced Immunochemical Studies, ShanghaiTech University, Shanghai, 201210 China; 4 Beijing Institute for Brain Disorders, Beijing, 100069 China

**Keywords:** Phosphatidylinositol 4-kinase IIα (PI4KIIα), Insulin secretion, Protein kinase D (PKD), Carriers of the trans-Golgi network to the cell surface (CARTS)

## Abstract

Insulin release by pancreatic β cells plays a key role in regulating blood glucose levels in humans, and to understand the mechanism for insulin secretion may reveal therapeutic strategies for diabetes. We found that PI4KIIα transgenic (TG) mice have abnormal glucose tolerance and higher serum glucose levels than wild-type mice. Glucose-stimulated insulin secretion was significantly reduced in both PI4KIIα TG mice and PI4KIIα-overexpressing pancreatic β cell lines. A proximity-based biotin labeling technique, BioID, was used to identify proteins that interact with PI4KIIα, and the results revealed that PI4KIIα interacts with PKD and negatively regulates its activity. The effect of PI4KIIα on insulin secretion was completely rescued by altering PKD activity. PI4KIIα overexpression also worsened glucose tolerance in streptozotocin/high-fat diet-induced diabetic mice by impairing insulin secretion. Our study has shed new light on PI4KIIα function and mechanism in diabetes and identified PI4KIIα as an important regulator of insulin secretion.

**Electronic supplementary material:**

The online version of this article (10.1007/s41048-018-0049-z) contains supplementary material, which is available to authorized users.

## Introduction

To maintain the balance of the glucose homeostasis, β cells of pancreas adapt their insulin secretory capability in response to various physiological and pathological demands (Zhang *et al.* 2012). The deterioration of insulin secretion can lead to the hyperglycemic environment that promotes loss of β cell mass and β cell dysfunction. Although insulin resistance has been received as the key character of type II diabetes (T2DM) for a long time, the development of obvious hyperglycemia requires a decrease in β cell function (Pimenta *et al.* 1995; Vauhkonen *et al.* 1998). β Cells are distinct endocrine cells that can respond positively by secreting insulin in response to changes of glucose concentration in the extracellular and to activators of phospholipase C, such as acetylcholine or cholecystokinin, and adenylate cyclase, such as glucagon, glucagon-like peptide-1, or gastric inhibitory polypeptide (Radosavljevic *et al.* 2004). The crucial regulators that can mediate glucose-stimulated insulin release are Ca^2+^, adenosine triphosphate (ATP), and diacylglycerol (DAG) (Radosavljevic *et al.* 2004; Rorsman and Renstrom 2003). In addition, there are many direct regulators of each step of insulin release, such as the packaging of insulin in small secretory granules, the trafficking of these granules to the plasma membrane, the exocytotic fusion of the granules with the plasma membrane, and the eventual retrieval of the secreted membranes by endocytosis (Easom 2000; Rorsman and Renstrom 2003). However, the regulation of insulin secretion is not precisely understood (Rorsman and Renstrom 2003).

Phosphatidylinositol kinases and phosphatidylinositol phosphates (PIPs) have recently been strongly associated with insulin secretion by pancreatic β cells. Researchers have indicated that phosphatidylinositol-4-phosphate (PI4P) and phosphatidylinositol-4,5-biphosphate [PI(4,5)P_2_] increase the insulin secretory response triggered by 10 μmol/L Ca^2+^, and insulin secretion was diminished by inhibiting the expression of type III PI4-kinase β (PI4KIIIβ) or type I phosphatidylinositol-4-phosphate 5-kinase γ (PI4P5Kγ) (Olsen *et al.* 2003; Waselle *et al.* 2005). Huang *et al.* showed that PI4P5Kα-knockout mice have increased first-phase insulin release and resist the high-fat diet (HFD)-induced development of type 2-like diabetes and obesity. In addition, they concluded that PI4P5Kα regulates insulin release from pancreatic β cells by helping maintain plasma membrane PI(4,5)P_2_ levels and the integrity of the actin cytoskeleton under both basal and stimulated conditions (Huang *et al.* 2011). Phosphatidylinositol-4-kinase IIα (PI4KIIα), the most abundant PI4K in mammalian cells (Balla and Balla 2006), localizes to the trans-Golgi network (TGN), endosomes and secreted vesicles and has been implicated in the regulation of protein sorting (Balla 2013; Guo *et al.* 2003; Minogue *et al.* 2006; Wang *et al.* 2003). Recently, Ketel *et al.* reported that depleting PI4KIIα causes defects in endosomal exocytosis and that PI(4)P produced by PI4KIIα on Rab11 endosomes is required for the recruitment of the exocyst to enable endosomal exocytosis (Ketel *et al.* 2016). Studies have also indicated that PI4KIIα is involved in recycling and retrograde transport (Jovic *et al.* 2014). Ryder *et al.* showed that PI4KIIα interacts with and regulates the WASH complex and influences vesicle transport (Ryder *et al.* 2013). In addition, PI4KIIα dysfunction contributes to several secretory diseases, such as breast cancer (Chu *et al.* 2010; Lang 2003; Li *et al.* 2010, 2014), spastic paraplegia (Simons *et al.* 2009), Gaucher’s disease (Jovic *et al.* 2012), and Alzheimer’s disease (Kang *et al.* 2013; Wu *et al.* 2004). However, nothing is known about PI4KIIα in diabetes, the most common disease associated with secretion.

In this study, we demonstrate that PI4KIIα transgenic (TG) mice have impaired glucose tolerance due to abolished insulin secretion under both physiological and pathological conditions. Mechanistic studies indicated that PI4KIIα influences insulin and CARTS complex secretion by regulating PKD activity. The above results suggest that PI4KIIα plays an important role in diabetes and insulin secretion.

## Results

### Generation and characterization of PI4KIIα TG mice

**Fig. 1:**
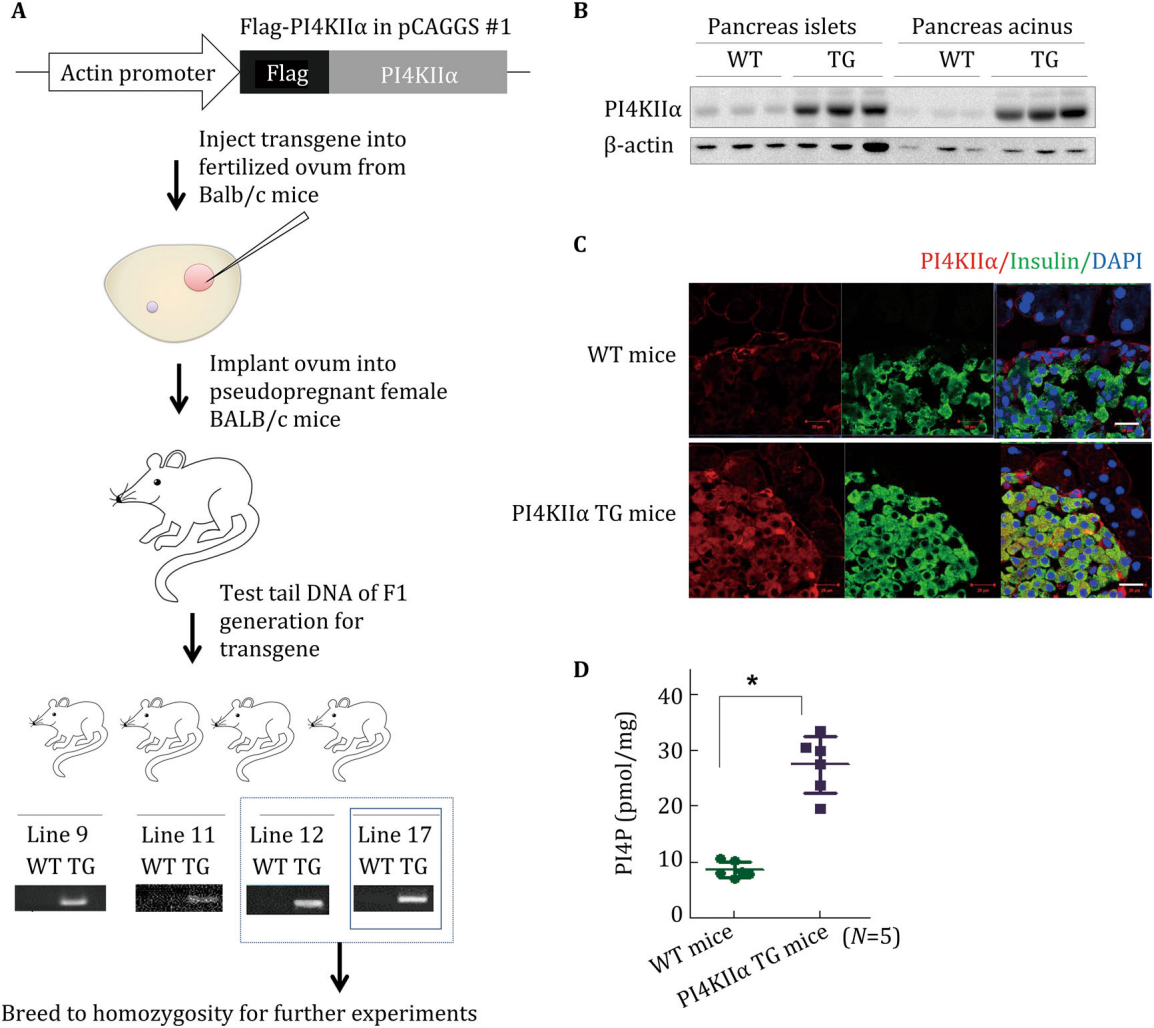
Generation of PI4KIIα transgenic (TG) mice. **A** Workflow to generate PI4KIIα TG BALB/c mice. **B** Islets and acinus were isolated from the mouse pancreas, and PI4KIIα expression was measured by Western blot. **C** The pancreases of WT and PI4KIIα TG littermates were sectioned at 10 µm using a cryostat. Protein expression in pancreas sections was determined using antibodies against insulin and PI4KIIα. Images were obtained using a laser confocal fluorescence microscope. Scale bar, 40 μm. **D** Islets were isolated from the pancreas of 25-week-old male PI4KIIα TG mice (line 17) and age-matched WT littermates (*N* = 5 for each line), and PI4P content was measured using a PI(4)P Mass Strip Kit. The data are presented as the mean ± SD. All the experiments except **A** were performed three times in triplicate. *p < 0.05

To study the function of PI4KIIα in T2DM and insulin secretion, we first investigated PI4KIIα expression levels in mouse models of diabetes. As shown in Supplementary materials (Figs. S1A, S1B), PI4KIIα expression in pancreatic islands was markedly increased in KK mice and *db/db* mice compared to wild-type (WT) C57BL/6 mice. These results demonstrate that PI4KIIα is upregulated with diabetes. To determine whether upregulated PI4KIIα expression plays a role in T2DM, a transgenic (TG) PI4KIIα-overexpressing BALB/c mouse model was generated (Fig. 1A). Four independent TG lines (lines 9, 11, 12, and 17) that expressed WT PI4KIIα protein were obtained. Lines 12 and 17 were chosen for further analysis (Fig. 1A). We then detected PI4KIIα expression levels by Western blot and found that it was upregulated in all detected tissues, including brain, pancreas, lung, stomach, fat, liver, intestine, spleen, heart, and muscle (Fig. S2A). Islet and acinar cells were isolated, and PI4KIIα expression was detected by Western blot. As shown in Fig. 1B, both islets and other neighboring cells in the pancreas expressed PI4KIIα, and immunohistochemistry studies indicated that PI4KIIα is highly colocalized with insulin-staining positive cells (Fig. 1C). Therefore, the data confirm that PI4KIIα is expressed in pancreatic β cells. We also analyzed the activity of overexpressed PI4KIIα; the PI4P content in the TG mouse pancreas was twofold higher than that in the WT mouse pancreas (Fig. 1D). PI4KIIα TG mice exhibited normal general health, viability, fertility, and body composition (data not shown). Although the TG mouse body weight was reduced (Fig. S2B), PI4KIIα overexpression had little effect on food intake (Fig. S2C) or plasma cholesterol, triglyceride, LDL-c, and HDL-c content compared to WT littermates (Fig. S2D).

### PI4KIIα overexpression abolishes glucose tolerance and insulin secretion

**Fig. 2:**
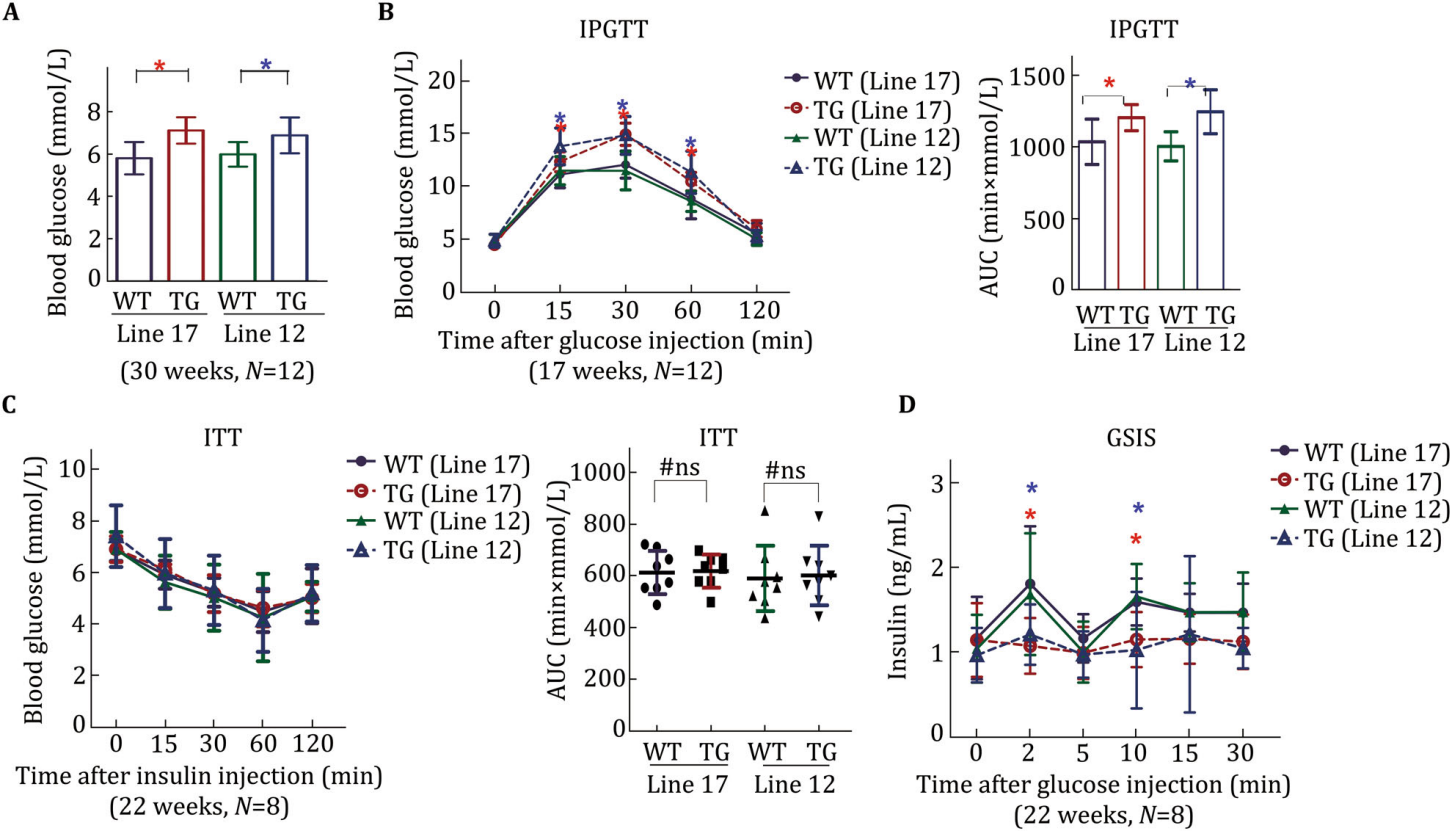
PI4KIIα overexpression impairs glucose tolerance and insulin secretion. **A** Fasting blood glucose was measured in 30-week-old male PI4KIIα TG mice (lines 12 and 17) and their respective WT littermates (*N* = 12 for each line). **B** Intraperitoneal glucose tolerance test (IPGTT) was performed in overnight-fasted 17-week-old male PI4KIIα TG mice (lines 12 and 17) and age-matched WT littermates (*N* = 12 for each line). **C** ITT was performed in 6-h fasted 22-week-old male PI4KIIα TG mice (lines 12 and 17) and age-matched WT littermates (*N* = 8 for each line). **D** GSIS was performed in overnight-fasted 22-week-old male PI4KIIα TG mice (lines 12 and 17) and age-matched WT littermates (*N* = 8 for each line). The data are presented as the mean ± SD. All the experiments were performed three times in triplicate. *p < 0.05

The above results indicated that PI4KIIα overexpression has no effect on the blood lipid profile. We then further tested its effect on blood glucose. Under normal chow-feeding conditions, PI4KIIα TG mice had slightly higher blood glucose levels (Fig. 2A) and impaired glucose tolerance (Fig. 2B) compared to their respective WT littermates after 16 h of fasting. However, insulin tolerance has no significant difference between PI4KIIα TG and WT mice, and the blood glucose level after insulin injection was not different among these four lines of mice (Fig. 2C), indicating no effect on insulin resistance. The observed results prompted us to evaluate the influence of PI4KIIα on insulin secretion. As shown in Fig. 2D, PI4KIIα overexpression significantly reduced insulin secretion during hyperglycemic stimulation; both the first and second phases were impaired in both lines (12 and 17) of PI4KIIα TG mice.

**Fig. 3:**
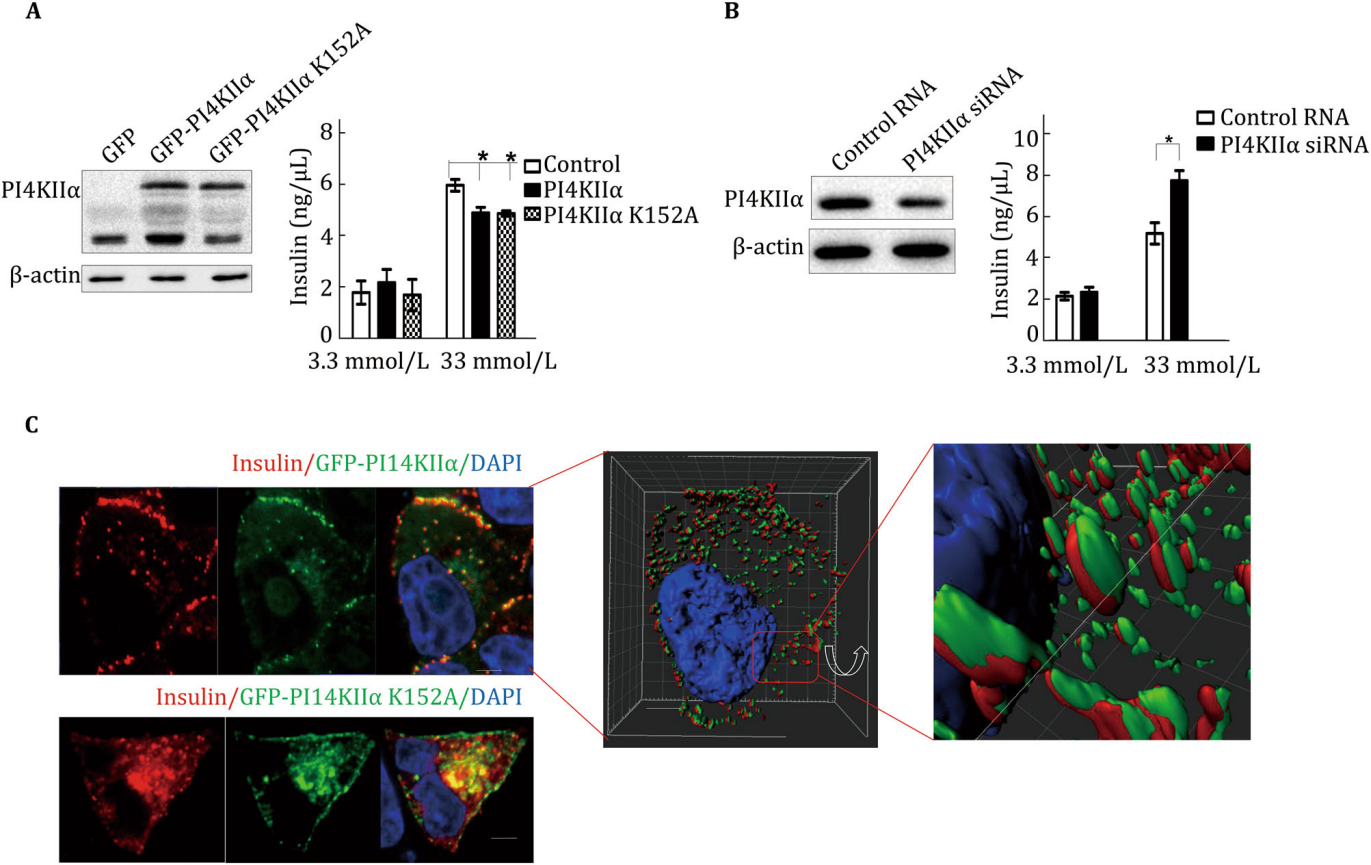
PI4KIIα regulates insulin secretion in MIN6 cells. **A** MIN6 cells overexpressing GFP, GFP-PI4KIIα, or GFP-PI4KIIα K152A. **B** MIN6 cells with siRNA-mediated knockdown of PI4KIIα. Insulin secretion in response to 3.3 or 33 mmol/L glucose was measured using an insulin ELISA kit. **C** Immunostaining of the nucleus (*blue*), insulin (*red*), and GFP-PI4KIIα or GFP-PI4KIIα K152A in MIN6 cells. The 3D cell model was built using Imaris software (Imaris 8 with colocalization; Bitplane, Belfast, UK). Scale bar, 2 μm. The data are presented as the mean ± SD of three independent experiments, and all the experiments were performed three times in triplicate. *p < 0.05

To further confirm that PI4KIIα regulates insulin secretion, we overexpressed GFP-PI4KIIα and the kinase-dead mutant GFP-PI4KIIαK152A (Minogue *et al.* 2006) in MIN6 cells (murine insulinoma-derived pancreatic β cell line), an insulin-secreting cell line (Ishihara *et al.* 1993). As shown in Fig. 3A, both WT and kinase-dead PI4KIIα reduced insulin secretion in response to high glucose (33 mmol/L) stimulation, indicating that PI4KIIα kinase activity is not necessary for its regulation of insulin secretion. Consistent with the above results, siRNA-mediated suppression of PI4KIIα expression in MIN6 cells significantly increased insulin secretion in response to stimulation with 33 mmol/L glucose (Fig. 3B), and this upregulation could not be rescued by adding PI4P, the product of PI4KIIα, to the cell culture (Fig. S3). These findings are consistent with the above result that overexpression of the kinase-dead PI4KIIα suppressed insulin secretion (Fig. 3A). Together, the results indicated that PI4KIIα can regulate insulin secretion independent of kinase activity. To study the regulatory mechanism, we first investigated whether pancreas islet mass was affected in PI4KIIα TG mice. As shown in Figs. S4A and B, neither islet size nor β cell mass were different between PI4KIIα TG mice and WT mice fed a normal chow diet. With the 4′,6-diamidino-2-phenylindole (DAPI), insulin, and BrdU triple staining of pancreatic sections, we discovered that there was no difference in BrdU incorporation into β cells between PI4KIIα TG mice and WT mice (Fig. S4C). Terminal deoxynucleotidyl transferase-mediated dUTP-biotin nick end labeling (TUNEL) assay results also indicated that PI4KIIα overexpression did not induce β cell apoptosis (Fig. S4D). Based on these results, we concluded that PI4KIIα overexpression reduced insulin secretion but did not affect β cell mass. We then questioned whether PI4KIIα directly regulates insulin secretion. To answer this question, we investigated the presence of insulin in PI4KIIα-positive granules by visualizing EGFP-tagged PI4KIIα and endogenous insulin. Insulin was juxtaposed with the PI4KIIα signal (Fig. 3C). A qualitative assessment was performed using more complex sections by viewing *Z*-stacks of images sequentially in a movie, which made it easier to follow particular structures in three dimensions (3D). Some of these *Z*-stacks were then converted to surface-rendered 3D objects by Imaris software (Imaris 8 with colocalization; Bitplane, Belfast, UK) to investigate spatial relationships and visualize colocalization. The results indicated that insulin was surrounded by PI4KIIα-containing organelles (Fig. 3C, Supplemental movie). All the above results indicated that PI4KIIα may regulate insulin secretion via protein–protein interactions.

### PI4KIIα regulates CARTS complex secretion

**Fig. 4:**
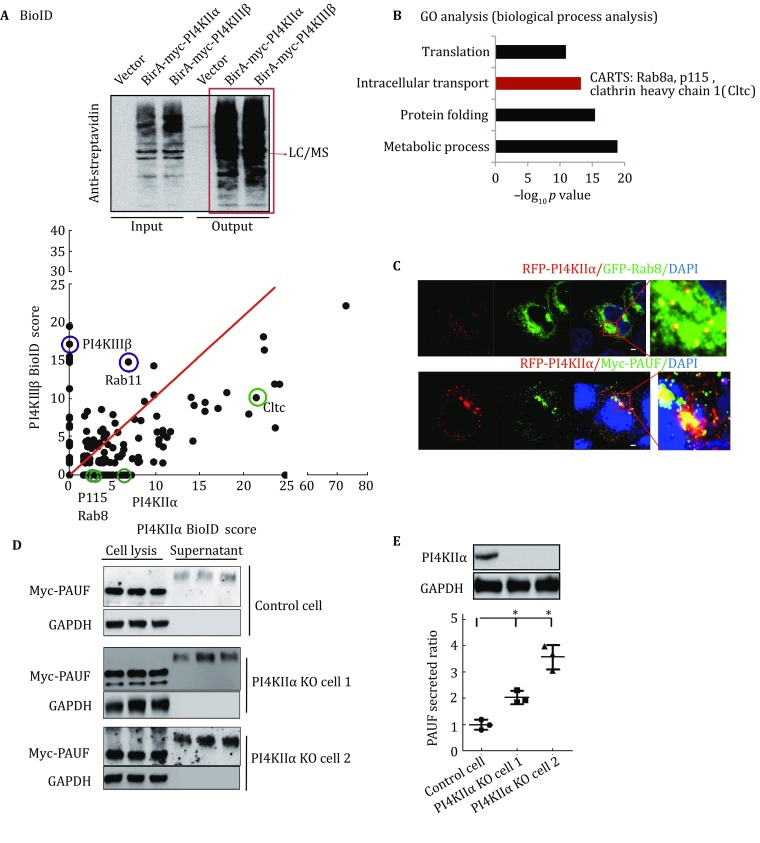
PI4KIIα interactome reveals its regulation of the CARTS complex. **A** Proteins biotinylated by BirA*-PI4KIIα or BirA*-PI4KIIIβ in MCF-7 cells were identified by mass spectrometry (LTQ-Orbitrap XL). **B** Gene ontology (GO) biological process analysis was performed to characterize these PI4KIIα proximate proteins. **C** RFP-PI4KIIα was cotransfected with GFP-Rab8 or Myc-PAUF into MCF-7 cells, and the nucleus (*blue*) or Myc-PAUF (*green*) were immunostained using DAPI or a Myc mouse monoclonal antibody, respectively. Scale bar, 2 μm. **D**, **E** Myc-PAUF was overexpressed in WT and PI4KIIα-knockout MCF-7 cells. After 30 h, cells were incubated in serum-free DMEM for another 8 h. Cell lysates and culture media were collected, PAUF and GAPDH were detected by Western blot (**D**), and the bands were analyzed using ImageJ (**E**). PI4KIIα expression levels were also detected by Western blot, with GAPDH as a control. The data are presented as the mean ± SD of three independent experiments, except for those from the LC–MS/MS experiments

We next checked a possible molecular mechanism by which PI4KIIα makes insulin exocytosis decline using BioID, which was an unbiased proteomic method and was developed for the characterization of protein–protein interaction networks recently. It was a kind of proximity-based biotin labeling (Roux *et al.* 2012). We ectopically expressed PI4KIIα fused to a mutant *Escherichia coli* biotin ligase (BirA R118G, or BirA*) in MCF-7 cells. BirA* efficiently activates biotin to label PI4KIIα proximate targets (Kwon *et al.* 2002). We used PI4KIIIβ as a control in this BioID experiment. All the hits are presented in Fig. 4A. We then analyzed these proximate proteins by Gene Ontology (GO) biological process analysis, and the result showed that proteins involved in translation, intracellular transport, protein folding, and metabolic process were enriched (Fig. 4B). Based on the observation that PI4KIIα can regulate insulin secretion, we then carefully analyzed the targets involved in intracellular transport, which are listed in Table 1. Interestingly, three individual components of the CARTS complex (Rab8a, p115, and clathrin heavy chain 1) (Wakana *et al.* 2012) are included in the PI4KIIα interaction target list but not that of PI4KIIIβ (Fig. 4A, B; Table 1). CARTS forms at the TGN and it is a class of transport carriers. Protein kinase D (PKD) is required for the trafficking of these carriers that contain Rab8a, p115, and a number of secretory and plasma membrane-specific cargos, such as pancreatic adenocarcinoma upregulated factor (PAUF) (Wakana *et al.* 2015). To verify the relationship between PI4KIIα and the CARTS complex, we ascertained the localization of PI4KIIα, an important component of CARTS (Rab8) and the most classical cargo of CARTS, PAUF, by immunofluorescence. As shown in Fig. 4C, PI4KIIα partially colocalized with both Rab8 and PAUF. Thus, we compared PAUF secretion in PI4KIIα-knockout cells and WT cells. Monoclonal MCF-7 knockout cell lines (Fig. S5) were generated by the CRISPR-CAS9 method. As shown in Fig. 4D and E, PAUF secretion was highly upregulated in both monoclonal PI4KIIα-knockout cell lines. These data are consistent with the finding that PI4KIIα suppression increases insulin secretion (Fig. 3B).

**Table 1:** Protein targets related to intracellular transport identified by LC–MS/MS in the PI4KIIα and PI4KIIIβ BioID experiments

Accession number	Description	Score 2α	Score 3β
P34058	Heat shock protein HSP 90-beta OS = *Rattus norvegicus* GN = Hsp90ab1 PE = 1 SV = 4—[HS90B_RAT]	51.98	35.20
P15999	ATP synthase subunit alpha, mitochondrial OS = *Rattus norvegicus* GN = Atp5a1 PE = 1 SV = 2—[ATPA_RAT]	41.16	16.54
P82995	Heat shock protein HSP 90-alpha OS = *Rattus norvegicus* GN = Hsp90aa1 PE = 1 SV = 3—[HS90A_RAT]	30.15	15.51
F1M779	Clathrin heavy chain 1 OS = *Rattus norvegicus* GN = Cltc PE = 2 SV = 1—[F1M779_RAT]	21.36	10.17
P62494	Ras-related protein Rab-11A OS = *Rattus norvegicus* GN = Rab11a PE = 1 SV = 3—[RB11A_RAT]	6.75	14.85
P46462	Transitional endoplasmic reticulum ATPase OS = *Rattus norvegicus* GN = Vcp PE = 1 SV = 3—[TERA_RAT]	6.57	0.00
Q99M64	Phosphatidylinositol 4-kinase type 2-alpha OS = *Rattus norvegicus* GN = Pi4k2a PE = 1 SV = 1—[P4K2A_RAT]	6.09	0.00
P09527	Ras-related protein Rab-7a OS = *Rattus norvegicus* GN = Rab7a PE = 1 SV = 2—[RAB7A_RAT]	5.19	1.62
B0BNK1	Protein Rab5c OS = *Rattus norvegicus* GN = Rab5c PE = 2 SV = 1—[B0BNK1_RAT]	4.31	1.77
Q63716	Peroxiredoxin-1 OS = *Rattus norvegicus* GN = Prdx1 PE = 1 SV = 1—[PRDX1_RAT]	3.98	0.00
Q4KM74	Vesicle-trafficking protein SEC22b OS = *Rattus norvegicus* GN = Sec22b PE = 1 SV = 3—[SC22B_RAT]	3.00	4.00
D4ABY2	Coatomer subunit gamma OS = *Rattus norvegicus* GN = Copg2 PE = 2 SV = 2—[D4ABY2_RAT]	2.49	0.00
G3V8T9	Apoptosis regulator BAX OS = *Rattus norvegicus* GN = Bax PE = 4 SV = 1—[G3V8T9_RAT]	1.88	0.00
P35280	Ras-related protein Rab-8A OS = *Rattus norvegicus* GN = Rab8a PE = 2 SV = 2—[RAB8A_RAT]	1.85	0.00
P41542	General vesicular transport factor p115 OS = *Rattus norvegicus* GN = Uso1 PE = 1 SV = 1—[USO1_RAT]	1.81	0.00
B2RYP4	Protein Snx2 OS = *Rattus norvegicus* GN = Snx2 PE = 2 SV = 1—[B2RYP4_RAT]	1.66	0.00
Q63413	Spliceosome RNA helicase Ddx39b OS = *Rattus norvegicus* GN = Ddx39b PE = 1 SV = 3—[DX39B_RAT]	0.00	3.95
B5DEP2	Protein Rab25 OS = *Rattus norvegicus* GN = Rab25 PE = 2 SV = 1—[B5DEP2_RAT]	0.00	1.70
O08561	Phosphatidylinositol 4-kinase beta OS = Rattus norvegicus GN = Pi4 kb PE = 1 SV = 1—[PI4KB_RAT]	0.00	17.18

### PI4KIIα-regulated insulin and CARTS secretion is dependent on PKD activity

**Fig. 5:**
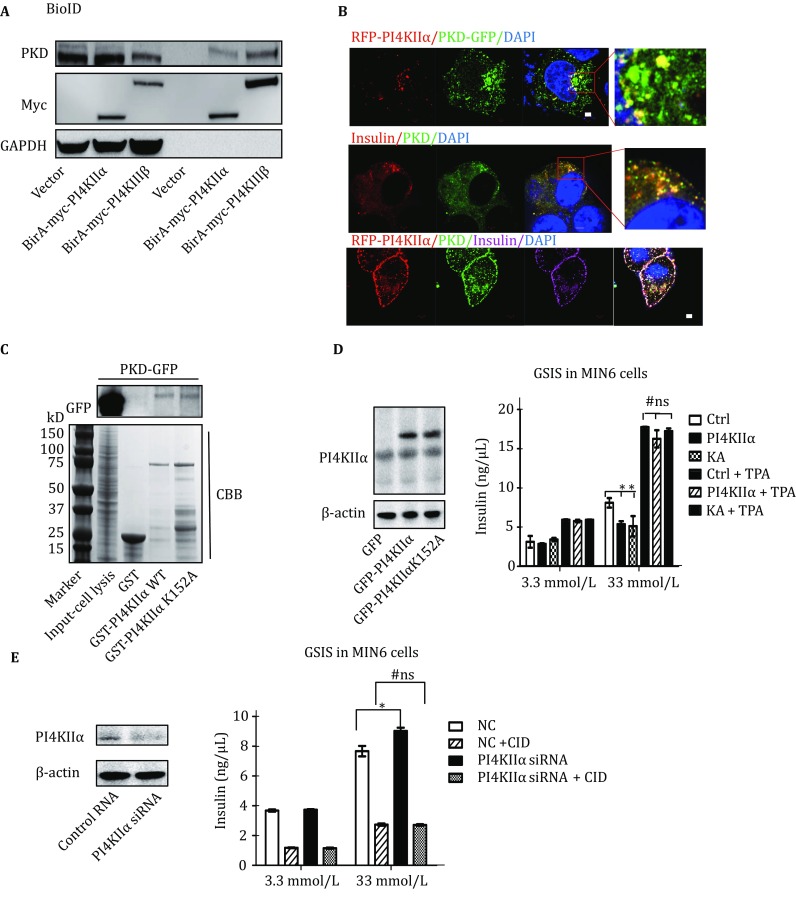
PI4KIIα regulates insulin secretion via a PKD-dependent pathway. **A** Proteins biotinylated by BirA*-PI4KIIα or BirA*-PI4KIIIβ in MCF-7 cells were purified using streptavidin agarose. The immunoprecipitates were immunoblotted with antibodies against PKD, Myc, and GAPDH. **B** MIN6 cells were transfected with or without RFP-PI4KIIα, and the nucleus (*blue*), PKD (*green*), and insulin (*red* or *magenta* as indicated) were immunostained using DAPI or the respective antibodies. Scale bar, 2 μm. **C** MCF-7 cells were transfected with GFP-PKD. After 24 h, the cells were lysed in RIPA buffer and subjected to pull-down with exogenous GST, GST-PI4KIIα WT, or GST-PI4KIIαK152A expressed in *E. coli*. **D** MIN6 cells were transfected with GFP, GFP-PI4KIIα, or GFP-PI4KIIαK152A. After 30 h, insulin secretion in response to 3.3 or 33 mmol/L glucose stimulation with or without 0.2 μmol/L TPA was measured using an insulin ELISA kit. **E** MIN6 cells were transfected with control siRNA or mouse PI4KIIα siRNA for 60 h; then, insulin secretion in response to 3.3 or 33 mmol/L glucose stimulation with or without 10 μmol/L CID755673 was measured using an insulin ELISA kit. The data are presented as the mean ± SD of three independent experiments, and all the experiments were performed three times in triplicate. *p < 0.05

PKD is essential for the biogenesis of the “TGN-to-cell-surface transport carriers” and is the most important common regulator of CARTS complex and insulin secretion (Sumara *et al.* 2009; Wakana *et al.* 2012). Therefore, we speculated that PI4KIIα-mediated regulation of insulin and CARTS secretion is dependent on the interaction between PI4KIIα and PKD. To address this hypothesis, we first determined whether PKD could be identified in a PI4KIIα BioID assay. As shown in Fig. 5A, PKD was labeled by both BirA*-PI4KIIα and BirA*-PI4KIIIβ; this finding is consistent with previous data showing that PI4KIIIβ is a substrate of PKD (Hausser *et al.* 2005). We then analyzed the colocalization of PI4KIIα, PKD, and insulin. As shown in Fig. 5B, both PKD and insulin colocalized perfectly with PI4KIIα in MIN6 cells. In addition, the interaction between PKD and PI4KIIα was confirmed by GST pull-down assay; human GFP-tagged PKD (GFP-PKD) was captured by both WT PI4KIIα and K152A-mutant PI4KIIα but not by the GST tag alone (Fig. 5C). This is consistent with what we observed previously, *i.e*., that both WT and kinase-dead (K152A) PI4KIIα reduce insulin secretion. To clarify whether this interaction contributes to the regulation of PKD activity, we evaluated that PKD activity in pancreatic islets isolated from WT mice or PI4KIIα TG mice. As shown in Fig. S6A, there was an obvious decrease in autophosphorylated PKD and phosphorylated PKD substrates in PI4KIIα TG mice. However, in PI4KIIα-knockout MCF-7 cells, PKD activity was markedly increased compared to that in WT MCF-7 cells (Fig. S6B). Thus, we can conclude that PI4KIIα regulates PKD activity via a protein–protein interaction. In addition, we performed rescue experiments to validate whether the regulation of insulin secretion by PI4KIIα is dependent on PKD activity. As shown in Fig. 5D, overexpression of either WT and kinase-dead (K152A) PI4KIIα obviously inhibited glucose-induced insulin secretion in MIN6 cells, while TPA (PKD agonist) treatment significantly increased insulin secretion. However, there was no difference in insulin secretion between WT MIN6 cells and PI4KIIα-overexpressing MIN6 cells upon TPA treatment. Consistent with this result, we observed that a PKD inhibitor (CID755673) blocked the PI4KIIα siRNA-mediated increasing of insulin secretion (Fig. 5E). The PKD inhibitor markedly reduced insulin secretion in response to stimulation with either low or high glucose, and PI4KIIα knockdown increased insulin secretion in only control MIN6 cells, not CID755673-treated cells. Together, these results indicated that the negative regulation of insulin secretion by PI4KIIα is dependent on PKD activity. To further confirm this regulatory pathway, we ascertained the effect of CID755673 on PAUF secretion induced by PI4KIIα knockout. As shown in Fig. S6C, inhibiting PKD activity obviously abolished the increased secretion of PAUF (traditional cargo for CARTS complex) induced by suppressing PI4KIIα. Based on these results, we concluded that PI4KIIα negatively regulates insulin and CARTS complex secretion and that this effect is dependent on PKD activity.

### PI4KIIα overexpression worsens glucose tolerance and insulin secretion in streptozotocin/high-fat diet-induced diabetic mice

**Fig. 6:**
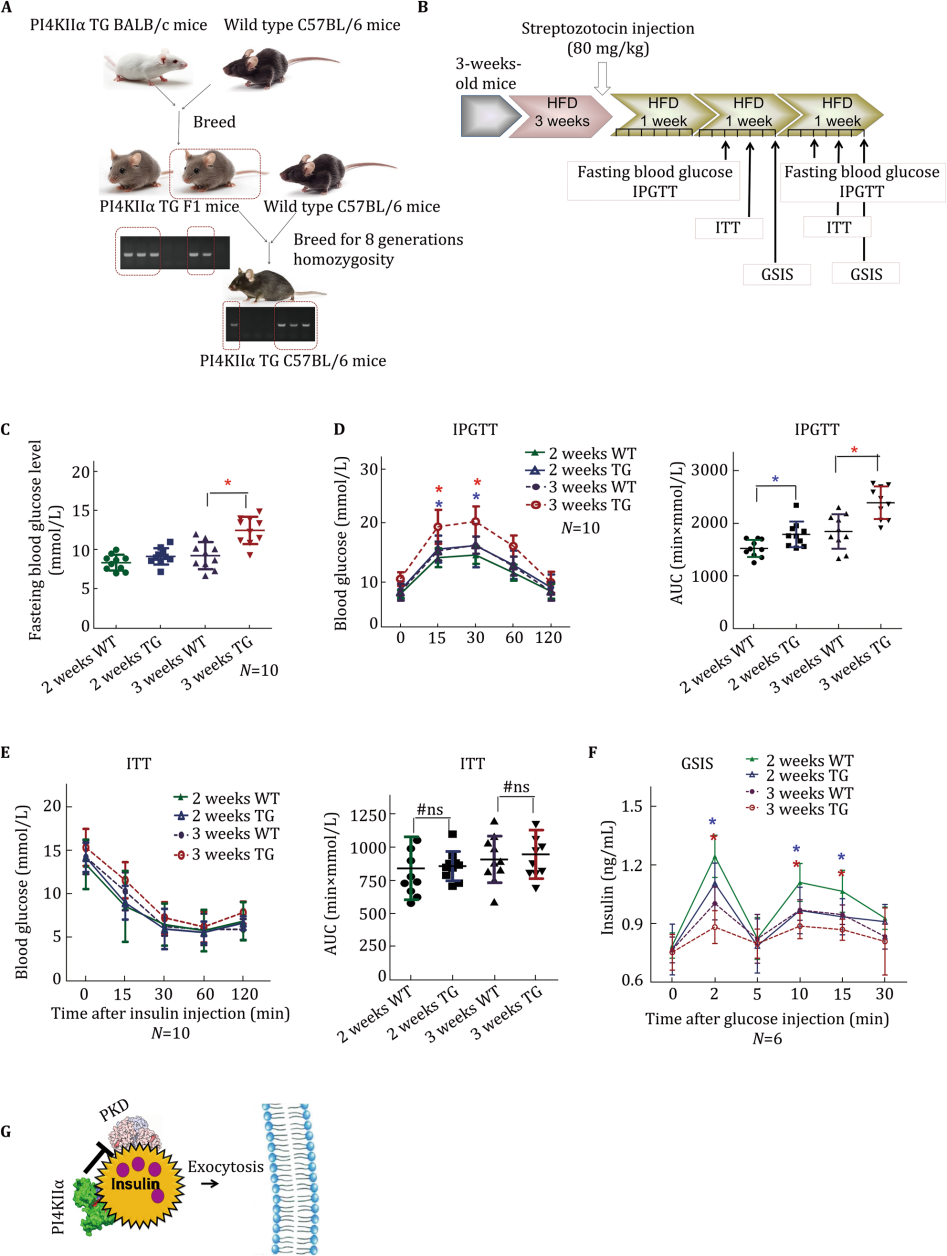
PI4KIIα overexpression enhances the sensitivity to STZ/HFD-induced diabetes in mice. **A** Workflow to generate PI4KIIα TG C57BL/6 mice from PI4KIIα TG BALB/c mice. **B** Workflow to generate diabetic mice by STZ/HFD treatment. **C** Fasting blood glucose was measured in STZ/HFD-induced mice. **D** IPGTT was performed in overnight-fasted PI4KIIα TG C57BL/6 mice and age-matched WT C57BL/6 littermates (*N* = 10 for each line). **E** ITT was performed in 6 h-fasted PI4KIIα TG C57BL/6 mice and age-matched WT C57BL/6 littermates (*N* = 10 for each line). **F** GSIS was performed in overnight-fasted PI4KIIα TG C57BL/6 mice and age-matched WT littermates (*N* = 6 for each line). **G** Hypothetic model: PI4KIIα can negatively regulate PKD activity via protein–protein interaction, while PKD activity is essential for insulin exocytosis. The data are presented as the mean ± SD. All the experiments were performed three times in triplicate. *p < 0.05

To determine whether PI4KIIα upregulation increases susceptibility to diabetes, we investigated the effect of a HFD and streptozotocin (STZ) treatment on PI4KIIα TG mice and WT littermates. BALB/c mice are insensitive to a HFD (Schreyer *et al.* 1998); thus, we first constructed PI4KIIα TG C57BL/6 mice by breeding PI4KIIα TG BALB/c mice with C57BL/6 WT mice, which are sensitive to a HFD. After 8 homozygous generations, PI4KIIα TG C57BL/6 mice were successful obtained (Fig. 6A). We first tested whether PI4KIIα overexpression increases serum glucose levels in C57BL/6 mice; fasting blood glucose level (Fig. S7A) and glucose tolerance (Fig. S7B) were impaired in PI4KIIα TG C57BL/6 mice, there was no significant difference in insulin tolerance (Fig. S7C), while insulin secretion was significantly weakened (Fig. S7D). These results were consistent with those in model mice on the BALB/c genetic background. Then, three-week-old male PI4KIIα TG and WT mice on the C57BL/6 genetic background were fed a HFD. After 3 weeks on a HFD, a single dose of STZ (80 mg/kg in 0.1 mol/L citrate buffer, pH 4.5) was administered by intraperitoneal injection. At 2 and 3 weeks after the injection, fasting blood glucose and glucose-stimulated insulin secretion (GSIS) were measured, and the intraperitoneal glucose tolerance test (IPGTT) and insulin tolerance test (ITT) were administered (Fig. 6B). The treatment highly raised serum glucose levels over time, and the STZ/HFD-induced hyperglycemic effect was extremely pronounced in the PI4KIIα TG mice (Fig. 6C). In addition, male PI4KIIα TG mice displayed relatively worse glucose tolerance after STZ/HFD treatment, with more rapid progression of diabetes compared to WT mice; the phenotype of PI4KIIα TG mice after 2 weeks was similar to that of WT mice after 3 weeks (Fig. 6D). Consistent with previous findings, the ITT results were not different between these two strains of mice (Fig. 6E), but PI4KIIα TG mice had lower glucose-induced insulin secretion compared to WT littermates (Fig. 6F). These results indicated that PI4KIIα TG mice are more sensitive to STZ/HFD treatment, and overexpressing PI4KIIα increased the susceptibility to diabetes.

## Discussion

Insulin secretion from pancreatic β cells is critical for the proper maintenance of blood glucose levels, and perturbations in this process lead to diabetes (Del Prato *et al.* 2002; Gupta *et al.* 2012). We provide new evidence that PI4Kα is a key regulator of β cell function in pancreas. The work of us uncovered a negative regulatory role for PI4Kα as shown in Fig. 6G of the hypothetic model: PI4KIIα can negatively regulate PKD activity via protein–protein interaction, while PKD activity is essential for insulin exocytosis.

PKD is a serine/threonine kinase that is activated by DAG signaling pathways to control fission and transport of Golgi vesicles, mediate survival responses to oxidative stress, regulate antigen-activated signaling in T and B cells, inhibit JNK-dependent proliferation, modulate adhesion, and elicit nuclear export of histone deacetylases (Ellwanger and Hausser 2013; Fu and Rubin 2011). Recently, researchers identified PKD as a pivotal regulator of stimulated insulin exocytosis (Sumara *et al.* 2009). In addition to its function in the TGN, PKD is thought to play an important role in priming insulin vesicles for transport and immediate fusion (Li *et al.* 2004; Sumara *et al.* 2009). Several studies have indicated that G protein-coupled receptor (GPR) 40 (Ferdaoussi *et al.* 2012; Iglesias *et al.* 2012) and MAPK p38δ (Sumara *et al.* 2009) influence insulin secretion by regulating PKD activity. Here, we revealed that PI4KIIα is a novel regulator of PKD activity by direct interaction, not by the DAG pathway (Figs. 5, S6). As shown in Fig. 5B, PKD and PI4KIIα colocalized at insulin-positive granules rather than at the TGN. Lu *et al.* showed that PKD localized at vesicular structures and promoted the recruitment of VAMP2 vesicles to the targeted membrane(Lu *et al.* 2007). Meanwhile, PI4KIIα was reported to have a similar function as PKD in regulating the association of VAMP3 with its cognate Q-SNARE Vti1a (Jovic *et al.* 2014). Therefore, we hypothesized that the PI4KIIα/PKD complex may have a role in the insulin and CARTS sorting process, which merits further investigation.

PI4KIIα is involved in various essential cellular functions, including membrane trafficking (Salazar *et al.* 2005; Wang *et al.* 2007, 2003), signal transduction (Li *et al.* 2010; Minogue *et al.* 2006; Pan *et al.* 2008), and the exo-endocytic cycle of synaptic vesicles (Guo *et al.* 2003). However, the precise mechanism of PI4KIIα in the cell is not yet completely deciphered because it engages in low-affinity interactions with dynamic cellular signaling pathways (Gokhale *et al.* 2016). Gokhale *et al.* identified novel interactors of PI4KIIα using a chemical cross-linker, DSP, combined with immunoprecipitation and immunoaffinity purification (Gokhale *et al.* 2016). Here, we used another transient and dynamic interaction method, BioID proximity-based biotin labeling, to identify proteins that interact with PI4KIIα. As shown in Table 1 and Fig. 4B, PI4KIIα participates in transient, low-affinity and dynamic interactions that are difficult to identify by direct pull-down or coimmunoprecipitation assays. This could explain that why the interaction between PI4KIIα and PKD identified by pull-down is quite weak; both proteins are highly dynamic in membrane trafficking and signal transduction (Balla and Balla 2006; Ellwanger and Hausser 2013). In addition, Rab8, Rab5, and Rab7 were also detected as PI4KIIα proximity targets (Table 1), which accord with previous results that PI4KIIα has an important role in late endosome (Salazar *et al.* 2005), early endosome, and sorting endosome (Henmi *et al.* 2016; Ketel *et al.* 2016) functions. Our results indicated that BioID could be an ideal tool for detecting dynamic PI4KIIα interactions and could provide valuable assistance in determining its functional role in physiologically and pathologically processes.

Recent studies indicated that PI4KIIα is essential for endosomal trafficking of transferrin and certain receptors (Henmi *et al.* 2016; Jovic *et al.* 2014; Ketel *et al.* 2016; Minogue *et al.* 2006). Therefore, we ascertained the effect of PI4KIIα knockout on transferrin recycling. As described by Jovic *et al.* (2014), suppressing PI4KIIα induced a significant delay in transferrin delivery to the recycling compartment (data not shown). Studies indicated that PI4KIIα is required for the production of endosomal PtdIns(4)P on early endosomes and for the sorting of transferrin and EGFR into the recycling and degradation pathways; both knocking down PI4KIIα and inhibiting its kinase activity influence the surface delivery of endosomal cargos (Henmi *et al.* 2016; Jovic *et al.* 2014; Ketel *et al.* 2016). However, in our study, we found that PI4KIIα is a negative regulator of insulin and PAUF secretion and that this regulation is completely independent of kinase activity: both WT and kinase-dead PI4KIIα reduced insulin secretion (Fig. 3A), and PI4P, the product of PI4KIIα, could not rescue the increase in insulin secretion upon PI4KIIα knockdown (Fig. S3). Together, the above results indicated that PI4KIIα has a different effect on different cargos, chiefly because of different regulatory mechanisms. The complexities of cargo classification and the intricate positive and negative feedback mechanisms among different cargos make it impossible to state an exact rule about the positive or negative regulation of various cargos by PI4KIIα; however, we will address this issue in the future.

To the best of our knowledge, this is the first study to reveal the pivotal role of PI4KIIα in regulating diabetes via insulin secretion and PKD. Our findings indicated that PI4KIIα is a new player in T2DM and that high PI4KIIα expression increases the susceptibility to HFD-induced hyperglycemia. Because PI4KIIα regulation of PKD and insulin secretion is independent of kinase activity, it is hard to evaluate its therapeutic effect in animal models of diabetes using inhibitors. However, the cellular assays indicated that suppressing PI4KIIα expression markedly increased insulin secretion (Figs. 3B, 5E). Therefore, it is worth developing tools to suppress PI4KIIα expression or disrupt the interaction between PI4KIIα and PKD and exploring the therapeutic effect against type 1 and type 2 diabetes; this will be the main direction of our future work.

## Materials and methods

### Reagents, plasmids, and antibodies

PI(4)P Mass ELISA Kit (K-4000E) was purchased from Echelon Biosciences. The original full-length human PI4KIIα plasmid was a kind gift from Shane Minogue (Minogue *et al.* 2001, University College London). pSpCas9(BB)-2A-GFP (PX458) (Addgene plasmid #48138) and lentiCRISPR V2 (Addgene plasmid #52963) were gifts from Feng Zhang (Ran *et al.* 2013). Antibodies to c-Myc, GAPDH, and β-actin were purchased from Santa Cruz Biotechnology (TX, USA). Antibodies to PKD, p-PKD (916), p-PKD (744/748), and PKD substrates were from Cell Signaling Technology (Herts, UK). Rabbit polyclonal PI4KIIα antibody was a kind gift from Pietro De Camilli (Guo *et al.* 2003, Yale University, HHMI). Insulin (Mouse) Ultrasensitive EIA was from Alpco (NH, USA). Other reagents were purchased from Sigma (Dorset, UK) unless otherwise stated.

### Generation of PI4KIIα transgenic mice

All animals were housed in the specific facilities which were pathogen-free and maintained on a 12-h light/dark cycle, and fed standard rodent chow at the Laboratory Animal Resources in the Institute of Biophysics, Chinese Academy of Science. Human PI4KIIα tagged with a flag epitope was subcloned into pCAGGS. The DNA was eluted in filtered microinjection buffer and injected into zygotes from BALB/c mice (purchased from Weitonglihua, Beijing, China). For genotyping, mouse tail DNA was isolated (by alkaline lysis) and analyzed by PCR (Forward primer: tctttcccgagcgcatctaccag; Reverse primer: agcagcaaggacagcacagcttc).

To study the function of PI4KIIα in STZ/HFD-induced diabetes, we generated PI4KIIα TG mice on the C57BL/6 genetic background. The first generation of heterozygous PI4KIIα TG mice was obtained by crossing WT C57BL/6 mice with PI4KIIα TG mice on the BALB/c genetic background; the resulting mice were the first (F1) generation. The identified PI4KIIα TG F1 mice were backcrossed with C57BL/6 WT mice for eight generations. Finally, we obtained heterozygous PI4KIIα TG mice on a pure C57BL/6 genetic background. At each generation, the genotype was confirmed by PCR.

### STZ/HFD-induced diabetic mouse model

Three-week-old male PI4KIIα TG mice (*N* = 10) on a C57BL/6 genetic background were fed a HFD (26.2% protein, 26.3% carbohydrate, 34.9% fat), and the control group (*N* = 10) comprised their WT littermates. After 3 weeks on a HFD, a single dose of STZ (80 mg/kg in 0.1 mol/L citrate buffer, pH 4.5) was administered by intraperitoneal injection to induce partial insulin deficiency. Three weeks after the STZ injection, the majority of animals fed a HFD and treated with STZ exhibited hyperglycemia. To monitor disease progression on STZ/HFD treatment, we tested fasting blood glucose, IPGTT, ITT, and GSIS at 2 and 3 weeks after STZ injection.

### BioID, on-bead protein digestion, and mass spectrometry

BioID was performed according to the previously described procedures (Roux *et al.* 2012). In brief, transfected cells were incubated with 50 µmol/L biotin for 6 h before harvest. Cells lysed as described above were incubated at 4 °C for 3 h with 500 µl of streptavidin conjugated to beads (New England Biolabs, Ipswich, MA). Beads were washed once with 1.5 ml of wash buffer 1 (2% SDS in H_2_O), once with wash buffer 2 (0.1% deoxycholate, 1% Triton X-100, 500 mmol/L NaCl, 1 mmol/L EDTA, and 50 mmol/L 171 HEPES, pH 7.5), once with wash buffer 3 (250 mmol/L LiCl, 0.5% NP-40, 0.5% deoxycholate, 1 mmol/L EDTA, and 10 mmol/L Tris, pH 8.1), and then twice with wash buffer 4 (50 mmol/L Tris, pH 7.4, and 50 mmol/L NaCl). To evaluate sample integrity, 10% of the total was retained for immunoblots. The remaining beads were centrifuged at 2000 *g* and resuspended in 50 µl of 50 mmol/L ammonium bicarbonate for mass spectrometry (LTQ-Orbitrap XL) as previously described (Roux *et al.* 2012).

### Islet isolation and Western blot analysis

Mouse islets were isolated by collagenase digestion of the pancreas according to previously described procedures (Martinez *et al.* 2006). In brief, overnight-fasted mice were anesthetized with an intraperitoneal injection of pentobarbital sodium (80 mg/kg body weight). The pancreas of mouse was inflated by the injection of 3 ml of a collagenase P solution (Sigma Chemical, St. Louis, MO; 0.5 mg/mL in Hank’s buffered salt solution). Pancreases were removed and incubated at 37 °C for approximately 20 min to make the digestion complete, which was stopped by the addition of 10 ml of Hank’s buffered salt solution containing 5% fetal bovine serum. The pancreases were washed three times with 10 ml of RPMI-1640 medium. Isolated islets were selected from the medium with the aid of a pipette under a stereoscopic microscope. The isolated islets were subjected to Western blot analysis or further incubated in RPMI-1640 with or without TPA (PKD agonist) at 37 °C for 30 min. Islets, cells or tissue were lysed and analyzed by Western blot using specific antibodies.

### Statistics

Statistical analysis was performed using the two-tailed paired Student’s *t* test. Differences were considered statistically significant at *p* < 0.05 or *p* < 0.01, as indicated in the legends. All data are presented as the mean ± SD.

## Electronic supplementary material

Below is the link to the electronic supplementary material.

Supplementary material 1 (AVI 12214 kb)

Supplementary material 2 (PDF 1248 kb)
