# Supplementary material for: PI4KIIα regulates insulin secretion and glucose homeostasis via a PKD-dependent pathway
Source: Biophys Rep. 2018 Mar 7;4(1):25–38. doi: 10.1007/s41048-018-0049-z (PMC5860104; doi:10.1007/s41048-018-0049-z)
Supplement: Supplementary file 2 — Supplementary material 2 (PDF 1248 kb) [file 41048_2018_49_MOESM2_ESM.pdf]

Supplementary materials

Cell culture, transfection and siRNA interference

MCF-7 cells and HEK293A cells were cultured in DMEM medium, INS-1 cells were cultured in RPMI Medium 1640 containing 11.1 mM glucose, MIN 6 cells were cultured in DMEM medium containing 25 mM glucose. And all above medium were supplemented with 10% fetal bovine serum, 55 mM β-mercaptoethanol and 100 units/mL penicillin, 100 μg/mL streptomycin, besides, INS-1 medium should be added sodium pyruvate to 1 mmol/L. And all cells were incubated in the presence of 5% CO_2 and 95% air at 37°C. Cells were transiently transfected with plasmids or siRNA using Lipofectamine 3000 reagent (Invitrogen) according to the manufacturer's instructions. For siRNA, the sequence targeting Rat PI4KIIα (GUGGAAGCUACUUCGUCAATT), mouse PI4KIIα (CAGCGGUCU-UCAAGUGAAUTT) were used. A non-targeting siRNA (*Thermotoga maritima* siRNA, UUCUCCGAACGUGUCACGUTT) was used as a negative control.

Mouse experiments

Mouse body weight and food intake were monitored at the same time on a weekly basis. For the GTT, 8-weeks-old male mice were fasted for 16 hours and then injected with glucose (2 g/kg body weight i.p.). Blood was drawn from the tail vein at 0, 15, 30, 60, and 120 minutes after glucose administration, and blood glucose level was measured with the Accu-Chek Aviva system from Roche. ITT was performed by injecting insulin (0.75 U/kg body weight i.p.) into 8-weeks-old *ad libitum*-fed male mice. Plasma glucose levels were measured as described above. GSIS was performed by injecting glucose (4 g/kg body weight i.p.). Blood was drawn from the tail vein at 0, 2, 5, 10, 15, 30, and 60 minutes after glucose administration. Serum insulin levels were determined using an ultrasensitive insulin enzyme immunoassay (Alpco Diagnostics).

β-cell proliferation assays

3-weeks-old male mice (n = 4/genotype) were injected intraperitoneally with 100 mg/kg of 5-bromo-29 deoxyuridine (BrdU; sigma) and killed 16 h after injection. Pancreases were isolated, fixed, and sectioned. Pancreas slices were analyzed by double staining with anti-BrdU (Millipore) and anti-insulin (Cell Signaling Technology) antibodies. Insulin-positive b-cells (2,500 β-cells/pancreas) were counted, and the percentage of BrdU- and insulin-double positive nuclei to total insulin-positive nuclei was determined for both TG mice and their WT control mice. Quantification of total BrdU-positive cell numbers was done by using an unbiased stereological method to ensure that the same BrdU-labeled cell is not counted twice

on adjacent sections and that the area of the pancreatic sections counted for each animal are consistent.

The analysis of islet mass and β-cell size

Male mouse pancreases were cut into 10 μm sections systematically through pancreatic head-to-tail axis, and sections were selected between every 200 μm. Five sections per mouse were picked. Mouse pancreatic slices were stained with an anti-insulin antibody. Images of pancreas slices were viewed by and captured Zeiss LSM710 microscope. All insulin-positive β-cell clusters (islets) were loosely traced, and the insulin-immunoreactive areas were determined by use of the threshold option. Total tissue areas were quantified with the threshold option to select the stained areas but not unstained areas (white space). The islet area (in square micrometers) and the area of each section were determined with Image J software (National Institutes of Health). Four to eight sections of each pancreas were covered by accumulating images from eight nonoverlapping fields of 1.5 × 106 μm^2. Analyses of β-cell area and size were performed using Image J software (National Institutes of Health). β-cell mass was calculated by insulin-positive area/total pancreas area times pancreas weight.

β-cell Transferase-mediated dUTP nick-end labeling assays

Male WT and TG mice (30-weeks-old) were killed, and pancreas weights of the mice were determined. Dewaxed paraffin sections of the pancreas were labeled with an *in situ* cell death detection kit (Roche) and hochest, and apoptosis was determined by immunofluorescence using a Zeiss LSM710 microscope. Four mice per group were used for transferase-mediated dUTP nick-end labeling (TUNEL) assays, and at least five islets (500–1,000 β-cells) were counted for each mouse.

Immunofluorescence of pancreas

The immunofluorescence experiments were performed as previously described1. In brief, pancreases obtained from 16-weeks-old male TG mice and respective WT littermates were cleared of fat and spleen, weighed and embedded in Optimal Cutting Temperature Compound (Ted Pella, Inc.). The tissues were cut into 10-μm sections using a microtome cryostat. The slices were fixed in ice-cold 4% paraformaldehyde (Sigma) and stained with specific antibodies.

Generation of PI4KIIα-knockout MCF-7 cells

PI4KIIα-knockout MCF-7 cells were generated using CRISPR/Cas9 technology. sgRNAs against human PI4KIIα were manually designed following published guidelines50 (PI4KIIα_CRISPR_Fw: 5’-GCCACTGTTGGATCGGGCCCG-3’) and

individually cloned into the PX458 vector; the resulting plasmids were transfected into recipient cells. Pools of PI4KIIα$^{-/-}$ cells were expanded from single cells. PI4KIIα knockout was verified by Western blot and immunofluorescence staining. Knockout cells were further confirmed by PCR sequencing (sense: ATGGACGAGACGAGCCCACTAG; antisense: CACCACCGCCTCGAACTCAG). We cloned the PCR product into the T vector. For each cell line, we selected 42 different individual TA clones for sequencing.

Insulin secretion assay in cell lines

Insulin content was detected as previously described52. Briefly, transfected INS-1 cells or MIN6 cells were washed three times with Krebs-Ringer bicarbonate HEPES buffer (KRBH, 119 mM NaCl, 4.6 mM KCl, 5 mM NaHCO3, 2.5 mM CaCl2, 1.2 mM MgSO4, 1.2 mM KH2PO4, 16 mM HEPES, and 0.1% BSA, pH 7.4). Following starvation by preincubation with KRBH buffer for 1 h, cells were incubated in KRBH in the presence of 3.3 or 33 mM glucose for 1 h. The supernatants were collected to measure insulin secretion using a Mouse Ultrasensitive Insulin ELISA kit according to the manufacturer's instructions.

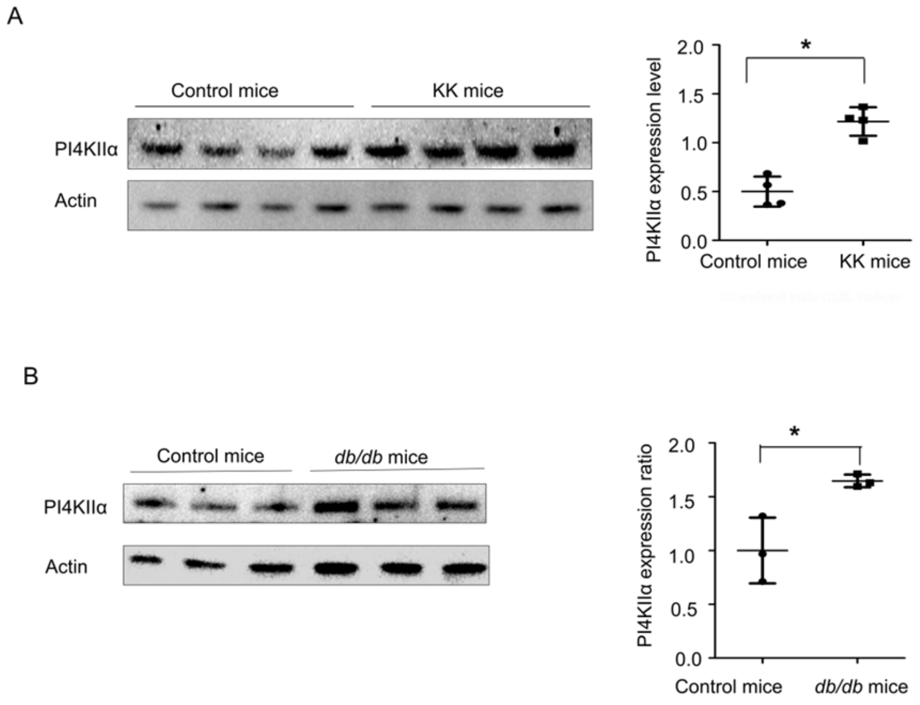

Fig. S1 PI4KIIα is upregulated in diabetic model mice. **A** Expression of PI4KIIα in KK mice and wild type C57BL mice by Western blot analysis (16-weeks-old male mice, $N = 4$). **B** Expression of PI4KIIα in *db/db* mice and wild type C57BL mice by Western blot analysis (13-weeks-old male mice, $N = 3$). Statistic analysis of PI4KIIα expression level in KK and *db*/*db* mice. The values are presented as the means ± S.D, $*p < 0.05$

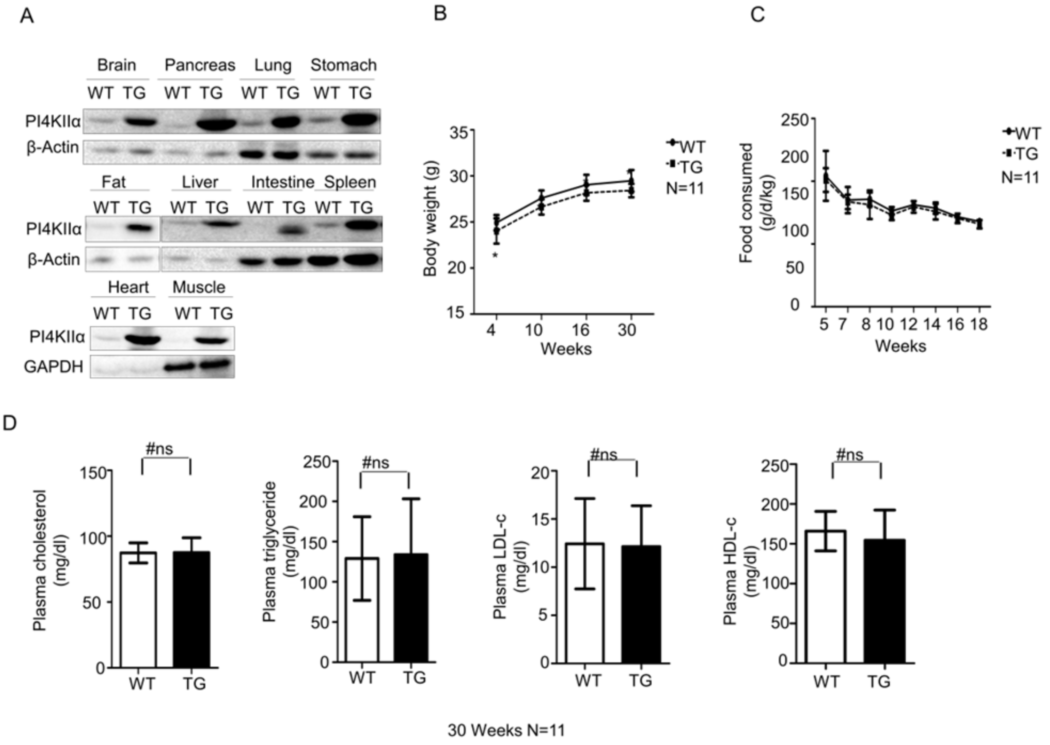

Fig. S2 Phenotype of PI4KIIα transgenetic mice. **A** Western blot analysis of tissues from 16-weeks-old male PI4KIIα TG (line 17) and wild type (WT) mice to confirm the successful transgenic of PI4KIIα in these tissues. Actin or GAPDH is shown as a loading control. Body weight **(B)** and food consumption (**C**) of PI4KIIα TG mice (line 17) and wild type mice were detected at indicated age (*N* = 11 for each line). **D** Plasma cholesterol, triglyceride, LDL-c and HDL-c were measured in 17-weeks-old male PI4KIIα transgenic mice (line 17) and age-matched WT littermates (*N* = 11 for each line). The values are presented as the means ± S.D. from three independent experiments, *p < 0.05, #ns means no significant difference

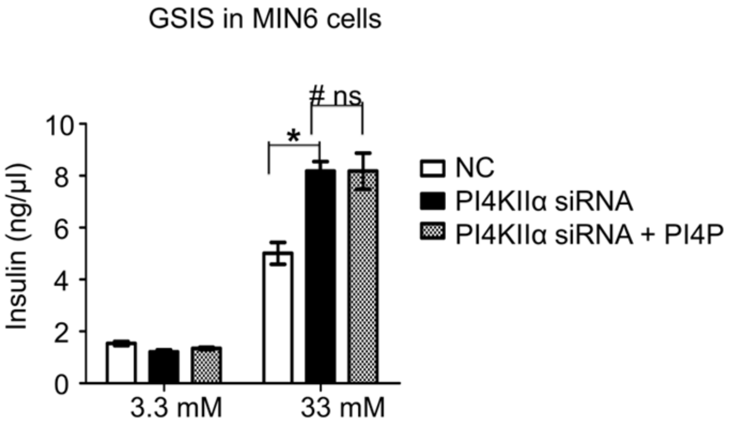

Fig. S3 PI4P cannot block the effect of PI4KIIα siRNA on insulin secretion. PI4P was pre-mixed with Carrier 3 (Echelon Biosciences Inc.) at 1:1 molar ratio for a final concentration of 500 μM at RT for 10 min and was added into the culture medium of the MIN6 cells transfected with control siRNA or rat PI4KIIα siRNA for 48 h, and the final concentration of PI4P was 50 μM. After 24-h incubation, the insulin secretion under 3.3 mM glucose and 33 mM glucose stimulation was measured by insulin ELISA kit. The values are presented as the means ± S.D. from three independent experiments, and all above experiments were performed three times in triplicate. *p < 0.05, #ns means no significant difference

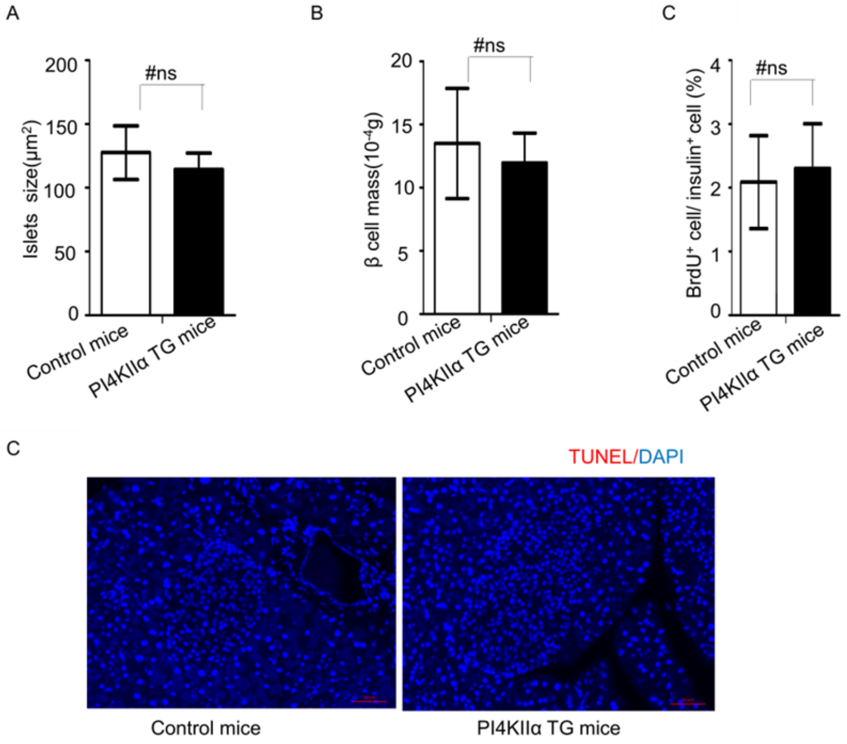

Fig. S4 PI4KIIα overexpression has no influence on mouse β cell mass, BrdU incorporation rate in βcells and β cells apoptosis. The islets area (**A**) and β cell mass (**B**) of PI4KIIα TG mice and wild type mice (30-weeks-old, *N* = 4 for each line) was measured using Image J. **C** β cell proliferation was determined by BrdU cell proliferation assay. BrdU was injected into 3-weeks-old male mice (100 mg/kg), and mice were killed 16 h after injection. More than 2,500 insulin-positive cell nuclei were counted per mouse under a microscope (*N* = 4 for each line). **D** The apoptosis of islets in PI4KIIα TG mice and wild type mice was detected by TUNEL. Nuclear (blue) or apoptosis cells (red) were immunostained by DAPI or TUNEL kit. The values are presented as the means ± S.D, #ns means no significant difference

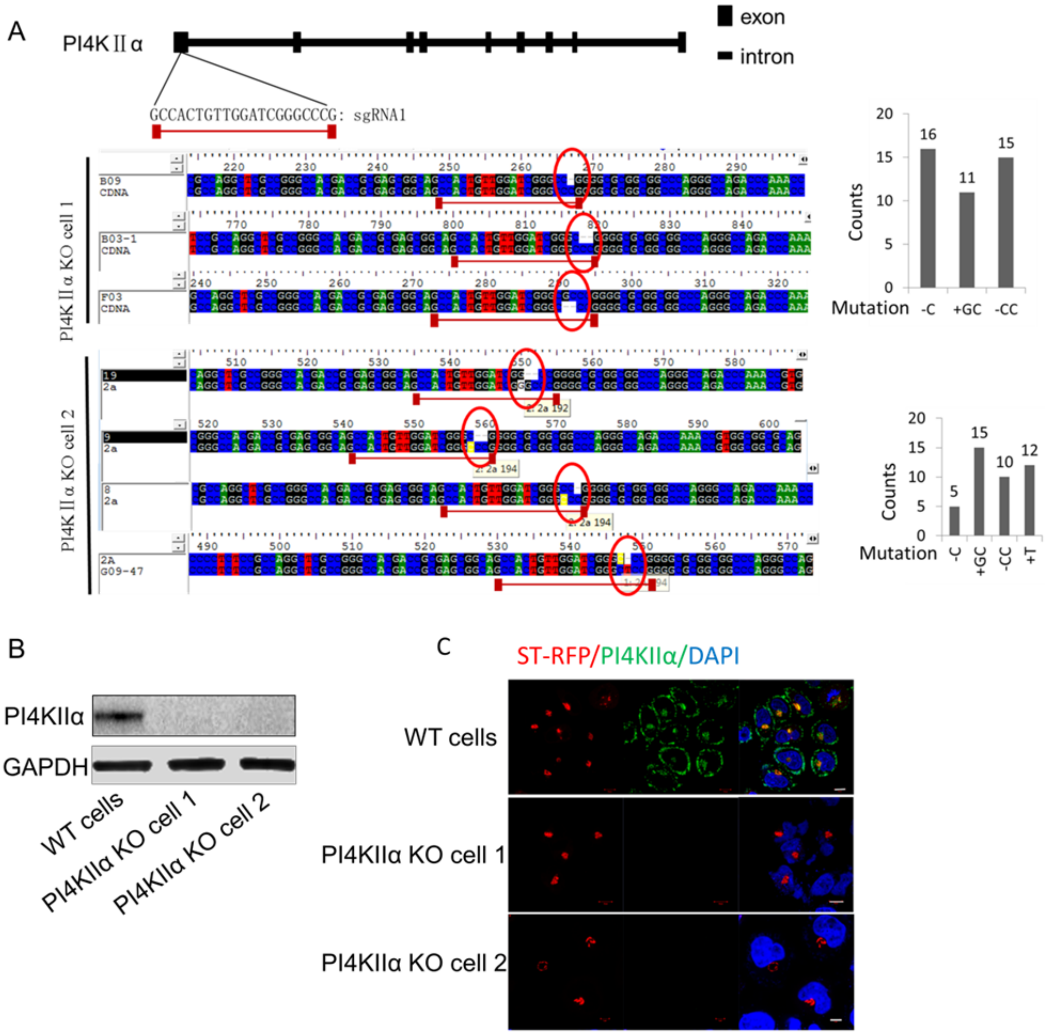

Fig. S5 Generate and identify PI4KIIα knock out monoclonal cell lines. **A** The sequence of PI4KIIα mutants in PI4KIIα knock out cell lines, sgRNA was labeled in red line. The sequence results were from 42 different TA clones for each cell line. The expression of PI4KIIα in wild type (WT) MCF-7 cell line and PI4KIIα knock out (KO1 and KO2) cell lines was measured by Western blot (**B**) and immunofluorescent staining (**C**). Experiments **B** and **C** were performed three times

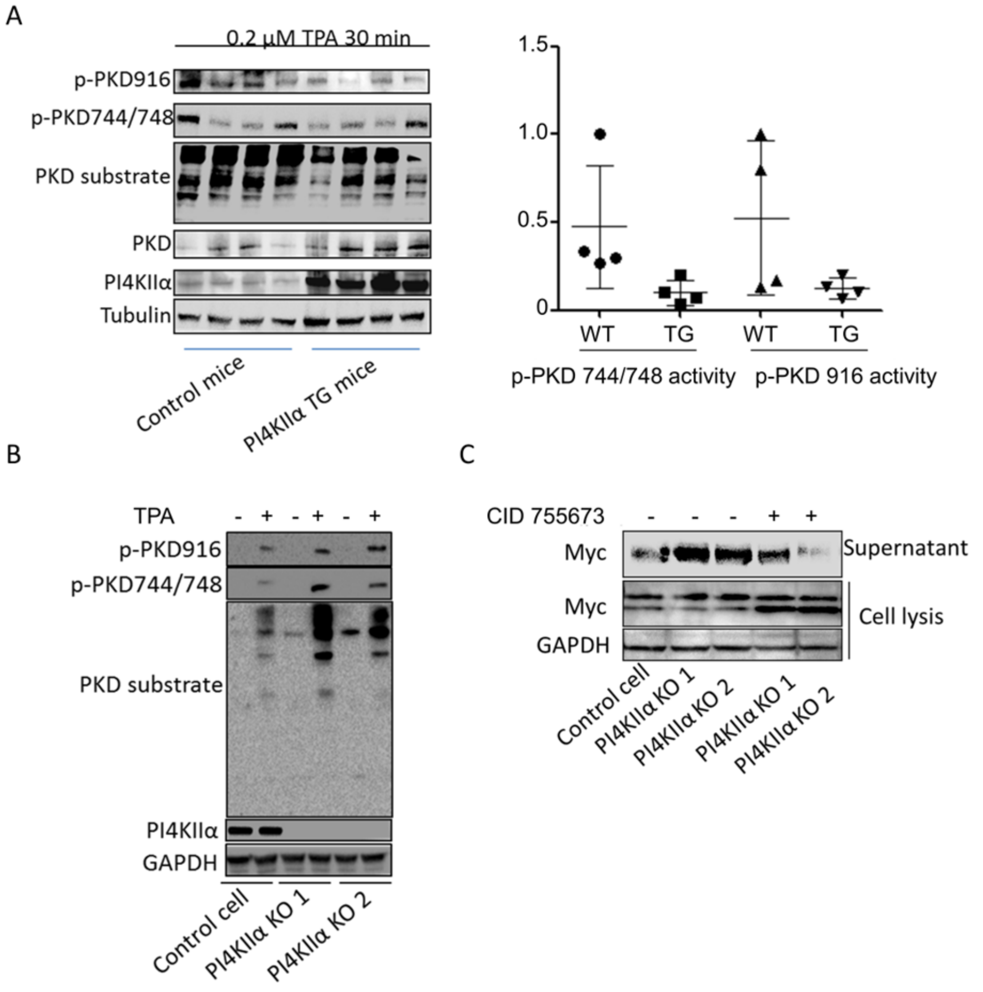

Fig. S6 PI4KIIα regulates PKD activity both *in vitro* and *in vivo*. **A** pancreatic islets were isolated from 22-weeks-old male PI4KIIα TG mice (line 17[#]) or age-matched WT littermates (*N* = 4 for each line), and then incubated in DMEM with 0.2 μM TPA for 30 min. The tissues were lysed by RIPA buffer and submitted for Western blot. The phosphorylated ratio of PKD was analyzed by Image J. The values are presented here as the means ± S.D. **B** Wild type MCF-7 cells and PI4KIIα knockout MCF-7 cells (PI4KIIα KO1 and PI4KIIα KO2) incubate with or without 0.2 μM TPA for 30 min, and indicated proteins were detected by Western blot. **C** Wild type MCF-7 cells and PI4KIIα knockout MCF-7 cells (PI4KIIα KO1 and PI4KIIα KO2) transfected with myc-PAUF for 24 h, and then incubate with or without 10 μM CID755673 serum free DMEM medium for another 8 h, cell culture medium and cell lysis were submitted for Western blot analysis. All above experiments were performed three times in triplicate

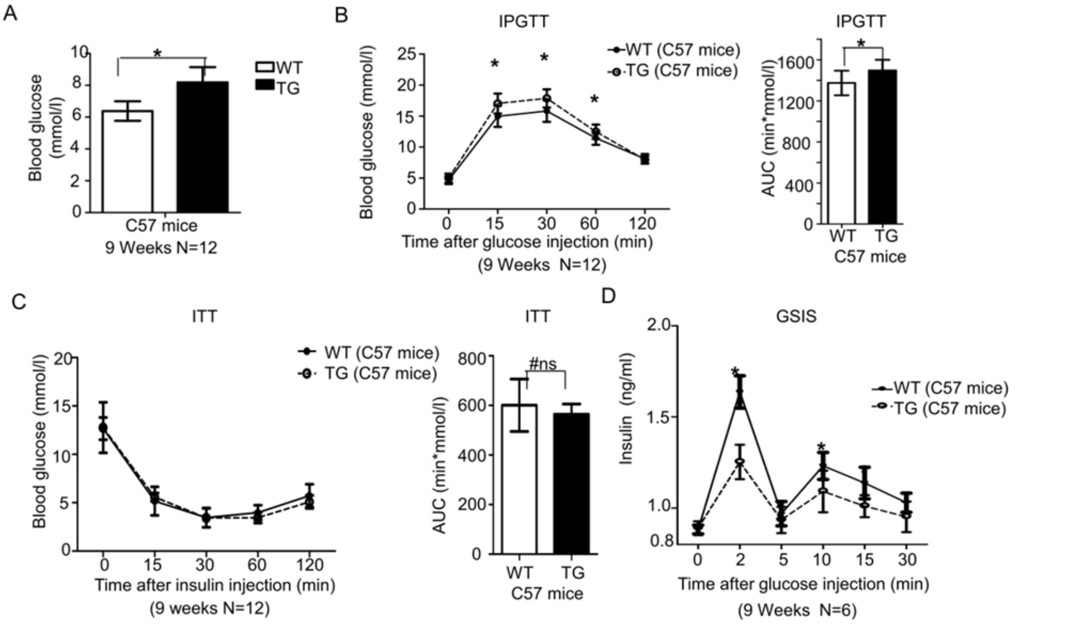

Fig. S7 PI4KIIα overexpression impaired glucose tolerance and insulin secretion in C57 mice. **A** Fasted blood glucose was measured in 9-weeks-old male PI4KIIα TG C57 mice and their WT littermates (N = 12 for each line). **B** IPGTT was performed in overnight fasted 9-weeks-old male PI4KIIα transgenic C57 mice and age-matched WT littermates (N = 12 for each line). **C** ITT was performed in 6-h fasted 9-weeks-old male PI4KIIα transgenic C57 mice and age-matched WT littermates (N = 12 for each line). **D** GSIS was performed in overnight fasted 9-weeks-old male PI4KIIα transgenic C57 mice and age-matched WT littermates (N = 6 for each line). The values are presented as the means ± S.D., and all above experiments were performed three times in triplicate. *p < 0.05, #ns means no significant difference

References：

1. Minogue, S. *et al.* Cloning of a human type II phosphatidylinositol 4-kinase reveals a novel lipid kinase family. *The Journal of biological chemistry* **276**, 16635-16640 (2001).
2. Ran, F.A. *et al.* Genome engineering using the CRISPR-Cas9 system. *Nature protocols* **8**, 2281-2308 (2013).
3. Shalem, O. *et al.* Genome-scale CRISPR-Cas9 knockout screening in human cells. *Science* **343**, 84-87 (2014).
4. Guo, J. *et al.* Phosphatidylinositol 4-kinase type IIalpha is responsible for the phosphatidylinositol 4-kinase activity associated with synaptic vesicles. *Proceedings of the National Academy of Sciences of the United States of America* **100**, 3995-4000 (2003).
5. Bernal-Mizrachi, E., Wen, W., Stahlhut, S., Welling, C.M. & Permutt, M.A. Islet beta cell expression of constitutively active Akt1/PKB alpha induces striking hypertrophy, hyperplasia, and hyperinsulinemia. *The Journal of clinical investigation* **108**, 1631-1638 (2001).
6. Hennige, A.M. *et al.* Upregulation of insulin receptor substrate-2 in pancreatic beta cells prevents diabetes. *The Journal of clinical investigation* **112**, 1521-1532 (2003).
7. Martinez, S.C., Cras-Meneur, C., Bernal-Mizrachi, E. & Permutt, M.A. Glucose regulates Foxo1 through insulin receptor signaling in the pancreatic islet beta-cell. *Diabetes* **55**, 1581-1591 (2006).
